# Adaptation and Assessment of a Text Messaging Smoking Cessation Intervention in Vietnam: Pilot Randomized Controlled Trial

**DOI:** 10.2196/27478

**Published:** 2021-10-08

**Authors:** Nan Jiang, Nam Nguyen, Nina Siman, Charles M Cleland, Trang Nguyen, Hue Thi Doan, Lorien C Abroms, Donna R Shelley

**Affiliations:** 1 Department of Population Health, Grossman School of Medicine, New York University New York, NY United States; 2 Institute of Social and Medical Studies Hanoi Vietnam; 3 Ronald O Perelman Department of Emergency Medicine, Grossman School of Medicine, New York University New York, NY United States; 4 Thai Nguyen University of Medicine and Pharmacy Thai Nguyen Vietnam; 5 Milken Institute School of Public Health, George Washington University Washington, DC United States; 6 School of Global Public Health, New York University New York, NY United States

**Keywords:** smoking cessation, text messaging, mHealth, mobile health, low- and middle-income country, smoking, developing countries, SMS, Vietnam

## Abstract

**Background:**

Text message (ie, short message service, SMS) smoking cessation interventions have demonstrated efficacy in high-income countries but are less well studied in low- and middle-income countries, including Vietnam.

**Objective:**

The goal of the research is to assess the feasibility, acceptability, and preliminary efficacy of a fully automated bidirectional SMS cessation intervention adapted for Vietnamese smokers.

**Methods:**

The study was conducted in 3 phases. In phase 1, we adapted the SMS library from US-based SMS cessation programs (ie, SmokefreeTXT and Text2Quit). The adaptation process consisted of 7 focus groups with 58 smokers to provide data on culturally relevant patterns of tobacco use and assess message preferences. In phase 2, we conducted a single-arm pilot test of the SMS intervention with 40 smokers followed by in-depth interviews with 10 participants to inform additional changes to the SMS library. In phase 3, we conducted a 2-arm pilot randomized controlled trial (RCT) with 100 smokers. Participants received either the SMS program (intervention; n=50) or weekly text assessment on smoking status (control; n=50). The 6-week SMS program consisted of a 2-week prequit period and a 4-week postquit period. Participants received 2 to 4 automated messages per day. The main outcomes were engagement and acceptability which were assessed at 6 weeks (end of intervention). We assessed biochemically confirmed smoking abstinence at 6 weeks and 12 weeks. Postintervention in-depth interviews explored user experiences among a random sample of 16 participants in the intervention arm.

**Results:**

Participants in both arms reported high levels of engagement and acceptability. Participants reported using the program for an average of 36.4 (SD 3.4) days for the intervention arm and 36.0 (SD 3.9) days for the control arm. Four of the 50 participants in the intervention arm (8%) reset the quit date and 19 (38%) texted the keyword TIPS. The majority of participants in both arms reported that they always or usually read the text messages. Compared to the control arm, a higher proportion of participants in the intervention arm reported being satisfied with the program (98% [49/50] vs 82% [41/50]). Biochemically verified abstinence was higher in the intervention arm at 6 weeks (20% [10/50] vs 2% [1/50]; *P*=.01), but the effect was not significant at 12 weeks (12% [6/50] vs 6% [3/50]; *P*=.49). In-depth interviews conducted after the RCT suggested additional modifications to enhance the program including tailoring the timing of messages, adding more opportunities to interact with the program, and placing a greater emphasis on messages that described the harms of smoking.

**Conclusions:**

The study supported the feasibility and acceptability of an SMS program adapted for Vietnamese smokers. Future studies need to assess whether, with additional modifications, the program is associated with prolonged abstinence.

**Trial Registration:**

ClinicalTrials.gov NCT03219541; https://clinicaltrials.gov/ct2/show/NCT03219541

## Introduction

Of the world’s 1.1 billion smokers, 80% live in low- and middle-income countries (LMICs) [1]. As a result, tobacco use is a major contributor to the high burden of noncommunicable disease and premature death in LMICs [2]. Promoting cessation is the key to reversing current global trends in tobacco-related morbidity and mortality over the next few decades [3].

Vietnam, an LMIC, has one of the highest smoking rates in the world [4]. According to the 2015 Global Adult Tobacco Survey, 45.3% of Vietnamese men were current smokers [5]. The country has implemented a range of evidence-based tobacco control policies as defined by the World Health Organization’s (WHO) Framework Convention on Tobacco Control including a national toll-free Quitline, launched in 2015 [6]. However, most smokers who attempt quitting do not call the Quitline or use cessation treatment [7]. In 2015, only 2.3% of recent quitters (who quit for less than 12 months) and current smokers who made past-year quit attempts received in-person or telephone treatment for smoking cessation [7].

To continue to meet goals for decreasing smoking prevalence globally, effective cessation interventions must be easily accessible, adapted to local languages and cultural contexts, and scalable. Mobile technology (mHealth) that uses text messaging or short message service (SMS) meets these criteria by creating a relatively low-cost platform for wide dissemination of tailored tobacco cessation interventions. A growing literature indicates that automated, bidirectional SMS cessation programs can be effective in increasing smoking cessation compared to minimal or no smoking cessation support [8-12]. However, this research has largely been conducted in upper middle-income countries [13]. The WHO Tobacco Free Initiative has emphasized the importance of developing mHealth solutions for increasing access to evidence-based tobacco cessation interventions in LMICs [14].

This study was conducted to address the gap in the literature by assessing the feasibility, acceptability, and preliminary efficacy of an SMS intervention for tobacco users in Vietnam. The study also provided an important opportunity to describe methods for adapting text message interventions found efficacious in high-income countries to different sociocultural contexts and forms of tobacco use.

Despite the large number of SMS studies conducted in high-income countries, few studies have compared the efficacy of combining SMS programs with additional cessation support to SMS alone, and findings have been mixed [12]. For example, Kruse et al [15] found that SMS combined with nicotine replacement therapy did not result in higher smoking abstinence compared with SMS alone. In contrast, the pilot study by White et al [16] found that combining SMS with personalized text message support from peer mentors increased cessation rates compared to SMS alone. Although there may be advantages to enhancing SMS interventions with additional support, this approach risks diminishing the potential cost advantage and scalability of automated SMS interventions [17]. Therefore, the goal of this study was to first adapt an SMS intervention to the sociocultural context of Vietnamese smokers and then compare the 6-week intervention to a control group that received a single text assessment on smoking status per week.

## Methods

### Study Design

The study was conducted in 3 phases. In the first phase, we conducted 7 focus groups (n=58 participants) to adapt text messages from SmokefreeTXT, a freely available public resource [18]. We supplemented that library with messages from Text2Quit to add topics not included in SmokefreeTXT like refusal skills and additional messages on harmful effects of tobacco use [19]. The second phase included a single-arm pilot test of the adapted SMS library with 40 participants. In the final phase, we conducted a 2-arm pilot randomized controlled trial (RCT) with 100 participants (98 males and 2 females). For all phases, eligible participants were (1) aged 21 to 55 years, (2) smoked ≥5 cigarettes per day (including dual users who used both cigarettes and waterpipe), (3) planned to quit smoking within the next 30 days, (4) had a mobile phone, (5) used text messaging in the past 6 months, and (6) lived in Hanoi, Vietnam. Exclusion criteria included current participation in other smoking cessation treatment and waterpipe-only users.

### Recruitment and Enrollment

We partnered with a community health center in Hanoi to recruit participants for each phase of the study. Community health collaborators, who are similar to community health workers and are assigned to work with the community health centers, were trained to disseminate study information through community outreach activities. During their routine outreach, they assessed smoking status of community members and shared study information with current tobacco users. If interested these individuals were asked for permission to share their contact information with research staff. Research staff then contacted potential participants to provide additional details, obtain consent, and enroll them in the study. This study was approved by the institutional review boards of New York University Grossman School of Medicine and Institute of Social and Medical Studies in Vietnam.

### SMS Adaptation Procedures

#### Conceptual Framework

The content of efficacious SMS interventions is based on a combination of several theoretical frameworks including social cognitive theory, transtheoretical model, and cognitive behavioral theory [8,19-23]. This includes the SMS libraries that we adapted for Vietnamese smokers [24,25]. These theories guided the design of the focus group and interview guides used in the formative data collection and message modifications. For example, in the prequit phase of the intervention, messages were designed to promote readiness to quit and increase motivation (eg, reasons for quitting), address outcome expectancies (eg, harms of smoking, benefits of quitting), reinforce self-efficacy, and offer advice for how to prepare for the quit date. In the postquit phase of the intervention, messages continued to offer motivational messages similar to those in the prequit phase but added an emphasis on the importance of obtaining social support and offered cognitive and behavioral strategies for dealing with social, emotional, and environmental triggers; coping adaptively with cravings; and resuming quit attempts after a slip or relapse.

#### SMS Intervention Adaptation

The final message library was developed through an iterative process that included first translating messages from the English language SMS libraries into Vietnamese with some initial changes to align the content to the Vietnamese context. For example, strategies for coping with nicotine cravings were edited to include practices that were relevant to Vietnamese smokers. We then conducted focus groups to assess message preferences; elicit suggestions to guide further adaptations; and assess reasons for and barriers to quitting, smoking triggers, and participants’ social networks and their influence on smoking behavior.

Focus groups included quantitative assessment of message preference followed by group discussions. Participants were asked to rate 46 text messages on a 1 to 4 scale (1=strongly dislike, 2=dislike, 3=like, 4=strongly like). Ratings were summarized while the focus groups elicited more details about smoking patterns and past quit attempts, reasons for quitting, and barriers to quitting. We then discussed a sample of the messages that were rated across the response scale options to gain additional insights about what types of messages were preferred and elicit suggestions for improving the messages.

Focus groups were moderated by two researchers and were audiorecorded, transcribed, and translated into English. Qualitative data analyses were conducted using NVivo 12 (QSR International). Using an inductive analytic approach, two research team members independently read a subset of transcripts (2-3) to identify preliminary themes, relevant patterns, and clustered concepts and generate questions [26,27]. Using an iterative process, the team continued to review transcripts until they reached consensus on a final codebook. One team member then coded the remaining transcripts.

Based on the findings from focus groups, the messages were further adapted. For example, compared to the original SMS programs, greater emphasis was placed on the impact of smoking on the family’s health and the dangers of smoking. Other content that was added to align with Vietnamese culture and specific tobacco use patterns included the health hazards of waterpipe use, which is still commonly used in Vietnam. Findings from focus groups also pointed to a need to develop additional messages that encouraged smokers to identify people in their network who could support their quit attempt and offered suggestions on how to refuse an offer of cigarettes or decline to smoke when others are smoking, a common scenario in a country with high male smoking rates (eg, “Think of your children when someone offers you to smoke... Tell them ‘I promised my children I wouldn’t smoke’”).

After finalizing the first draft of the SMS library, we enrolled 40 participants in a single-arm pilot test. The participants received automated bidirectional text messages for 6 weeks. At the end of the pilot test, two researchers conducted in-depth interviews with a random sample of 10 participants. The interviews explored 5 areas:

- Overall perceptions about the program (eg, “What did you think of the program?”)
- Perceptions about specific program features such as the opportunity to type in the keyword TIPS to obtain additional advice on how to deal with cravings
- Perceptions about specific message themes (eg, “What types of messages were most helpful? Which were least helpful?”). We read some text messages that participants received during the intervention and asked what they liked and didn’t like about the messages, as well as their suggestions (eg, “How can we improve the messages to make them more helpful for you?”)
- Perceptions about other program characteristics including the number and timing of text messages and length of the program
- Suggestions for improving the SMS program.

Similar to the focus groups, the in-depth interview was guided by our integrated conceptual framework. Two researchers moderated and audiorecorded the interviews. Interviews were transcribed and translated into English. Two researchers used the same approach used to analyze the focus group data. Findings from the single-arm pilot test demonstrated the feasibility of retaining participants in the 6-week SMS cessation intervention and informed additional modifications to the content of the message library. These included a greater emphasis on harms of smoking versus benefits of quitting and adding more messages that offered concrete advice about coping with cravings rather than vague messages meant to motivate smokers (eg, “Stay strong, you can do it”).

### RCT Procedures

We conducted the pilot RCT (Multimedia Appendix 1) between November 2018 and March 2019 with 100 participants including 98 men and 2 women. Participants provided written consent at the time of enrollment and were randomized to the intervention (n=50) or control arm (n=50) using block randomization stratified by cigarette consumption per day (CPD; 5-10 vs >10 CPD). Participants completed a baseline survey at enrollment and follow-up surveys at 6 weeks and 12 weeks postenrollment. All surveys were administered in person by a research assistant. At the end of the intervention period, we conducted in-depth interviews with a random sample of 16 participants from the intervention arm to obtain more in-depth information about their experiences with the program. Participants were compensated for text messaging charges that occurred during the intervention and received VND 50,000 (US $2.23) for each survey and VND 100,000 (US $4.46) for the in-depth interview.

### SMS Intervention

The final message library consisted of 188 text messages. Messages were designed to increase knowledge about smoking and motivation to quit (elicit reasons for quitting, describe harms of smoking and secondhand smoke exposure and harms of waterpipe use, provide information about the Quitline); change outcome expectancies (benefits of quitting); and offer cognitive and behavioral strategies such as refusal skills to assist smokers in maintaining the quit attempt. Behavioral strategies encouraged self-efficacy for quitting and encouraged smokers to obtain social support from family and friends.

The intervention consisted of a 2-week prequit period and a 4-week postquit period. Participants received 2 or 3 messages per day during the 2-week prequit period, 4 on the quit date, 3 or 4 per day during the first 2 weeks after the quit date, and 2 or 3 messages per day during the subsequent postquit period. Prequit messages encouraged smokers to track their smoking behavior and identify triggers, reinforced reasons for quitting, elicited smokers’ reasons for quitting, and provided advice on obtaining social support as they neared their quit date. Postquit messages were oriented toward relapse prevention and maintaining motivation and included the themes described above. In addition to programmed outgoing messages, participants could send the keyword TIPS to the program to trigger on-demand messages for additional support.

Starting from the quit date, participants received a weekly bidirectional text message to assess smoking status as follows: “Are you smoke-free? Reply: Yes or No.” Those who responded yes continued to receive postquit messages. Those who answered no received a message asking if they preferred to set a new quit date. Those who responded no continued to receive postquit messages, and those answering yes received a call from the research assistant to obtain a new quit date and reset the quit date in the SMS program which returned to the prequit protocol. In addition, participants received bidirectional text messages that assessed their level of craving (hi, med, low) on days 1, 3, 5, 8, 15, and 25. A high or medium craving response triggered an automated message offering TIPS from the SMS program. Participants could opt out of the SMS program at any time by texting STOP. At the start of the program, participants were made aware that they had the option to text STOP at any time during the trial to discontinue receiving messages.

### Control Arm

Participants in the control arm received one text assessment message per week at a fixed time in the evening during the 6-week intervention period: “Are you smoke-free? Reply: Yes or No.” The control condition was consistent with previous SMS cessation trials that included minimum exposure for participants in the control group [15,28].

### Measures

We conducted surveys at baseline and at 6 weeks (end of treatment) and 12 weeks. The baseline survey captured sociodemographic information such as gender, age, education, and household income level; text messaging habits; and smoking behavior, including CPD and waterpipe sessions per day. Participants were dichotomized as dual users if they reported waterpipe use on some day or every day or cigarette-only smokers if they responded not at all to the waterpipe use question.

Measures of feasibility included reach (ie, the proportion of individuals approached who enrolled) and survey assessment response rates. We also tracked if participants experienced technical problems.

Two measures of program engagement were assessed using the 6-week survey: (1) the number of weeks that participants reported using the program (calculated as the mean number of days using the program based on that response) and (2) self-reported frequency of reading the messages (always/usually/sometimes/never). Two additional measures included the proportion of participants who responded to the bidirectional text message assessments with mean number of times they responded and the proportion who texted the keywords (eg, TIPS) with mean number of times they texted the keyword.

Program acceptability was assessed at 6 weeks by asking participants to rate their overall satisfaction with the SMS program (for intervention arm) or the weekly text assessment (for control arm; very satisfied/satisfied/unsatisfied/very unsatisfied), perceived number of messages (too many/just right/too few), and their agreement with statements such as “The text messages helped me quit smoking” using a 4-point Likert scale from “strongly disagree” to “strongly agree.”

Smoking abstinence was assessed at 6 weeks and 12 weeks. Abstinence was defined as self-reported no smoking in the past 7 days confirmed with a carbon monoxide of 10 ppm or less [29]. Quit attempts were assessed by asking participants if they had ever stopped smoking cigarettes for a day or more during the intervention period because they were trying to quit (yes/no). Reductions in cigarettes smoked per day was calculated as the difference in CPDs at baseline compared with 6 weeks and 12 weeks.

### In-Depth Interview Procedures

We conducted in-depth interviews with 16 randomly selected participants from the intervention arm. Using a semistructured guide similar to the single-arm pilot test, two researchers moderated and audiorecorded the interviews to obtain more in-depth information about what they liked and didn’t like about the program and elicit recommendations for improving the SMS program. Interviews were transcribed and translated into English.

### Data Analysis

We analyzed quantitative data using the R statistical computing environment (R Foundation for Statistical Computing) [30]. Sociodemographic characteristics of the sample, the intention-to-treat abstinence rates, and other cessation outcomes were compared by study arm using Pearson chi-square and *t* tests. We used descriptive statistics to summarize program acceptability and engagement results. All tests of statistical significance were 2-tailed, and *P*<.05 was considered significant. The process for qualitative analyses was the same across the 3 aims and described above.

## Results

### Participant Demographics and Smoking Behavior

On average, participants were aged 38.9 (SD 8.2) years (Table 1). A total of 77.0% of participants (77/100) graduated from high school or had attended vocational school or college, and 70.0% (70/100) had a household income level of more than VND 100,000,000 (US $4,455.5). Our sample included more cigarette-only smokers (58/100, 58.0%) than dual users (42/100, 42.0%). Participants smoked an average of 15.4 (SD 8.2) CPD. Dual users reported a mean of 11.8 (SD 10.4) waterpipe sessions per day.

**Table 1:** Sociodemographic and tobacco use characteristics of participants at baseline by study arm.

Characteristic		Total (n=100)	Intervention arm (n=50)	Control arm (n=50)	*P* value
Age (years), mean (SD)		38.9 (8.2)	40.0 (7.5)	37.7 (8.7)	.17
**Educational attainment, n (%)**		—^a^	—	—	.63
	Primary school or less	2 (2.0)	2 (4.0)	0 (0)	—
	Middle school	21 (21.0)	10 (20.0)	11 (22.0)	—
	High school	38 (38.0)	20 (40.0)	18 (36.0)	—
	Vocational school or college	39 (39.0)	18 (36.0)	21 (42.0)	—
**Household income level, n (%)**		—	—	—	.12
	<50,000,000 VND	4 (4.0)	3 (6.0)	1 (2.0)	—
	50,000,000-100,000,000 VND	24 (24.0)	15 (30.0)	9 (18.0)	—
	>100,000,000 VND	70 (70.0)	30 (60.0)	40 (80.0)	—
	Unreported	2 (2.0)	2 (4.0)	0 (0)	—
**Type of smoker, n (%)**		—	—	—	.31
	Cigarette-only smoker	58 (58.0)	32 (64.0)	26 (52.0)	—
	Dual user	42 (42.0)	18 (36.0)	24 (48.0)	—
Cigarette consumption per day, mean (SD)		15.4 (8.2)	15.4 (8.0)	15.4 (8.6)	.98
Number of waterpipe sessions per day^b^, mean (SD)		11.8 (10.4)	14.3 (12.9)	9.9 (7.7)	.18
**Cigarette quit attempt in the past 12 months, n (%)**		—	—	—	.41
	Yes	36 (36.0)	34 (68.0)	30 (60.0)	—
	No	64 (64.0)	16 (32.0)	20 (40.0)	—

^a^Not applicable.

^b^Among dual users only (n=42).

### Feasibility

Almost all of those screened were eligible and 99.0% (100/101 eligible participants) enrolled in the study. All participants completed the 6-week and 12-week follow-up surveys. There were no technical issues reported by participants or the SMS vendor.

### Engagement

The mean number of days that participants reported using the program was 36.4 (SD 3.4), out of a total of 42 days, in the intervention arm and 36.0 (SD 3.9) for the control arm (Table 2). None of the participants texted the keyword STOP to unsubscribe from the program. Among participants in the intervention arm, 8% (4/50) reset the quit date and 38% (19/50) texted the keyword TIPS to trigger on-demand messages at least once (only the intervention arm has these options). The majority of participants in both arms reported that they always or usually read the text messages.

**Table 2:** Participant engagement and program acceptability.

Measure				Intervention arm (n=50)		Control arm (n=50)
**Engagement**						
	Number of days used program^a^, mean (SD)			36.4 (3.4)		36.0 (3.9)
	Ever texted TIPS to the SMS program, n (%)			19 (38)		—^b^
	Mean number of times texted TIPS^c^, mean (SD)			5.1 (8.1)		—
	Ever responded to text assessment, n (%)			36 (72)		—
	Mean number of times responded to text assessment^d^, mean (SD)			5.3 (4.1)		—
	**Frequency of reading messages, n (%)**					
		Always	27 (54)		18 (36)	
		Usually	14 (28)		22 (44)	
		Sometimes	9 (18)		10 (20)	
		Never	0 (0)		0 (0)	
**Acceptability**						
	**Overall satisfaction with the program^a^, n (%)**					
		Very satisfied	14 (28)		0 (0)	
		Satisfied	35 (70)		41 (82)	
		Unsatisfied	1 (2)		0 (0)	
		Very unsatisfied	0 (0)		9 (18)	
	**Number of messages received from the program^a^, n (%)**					
		Too many	11 (22)		3 (6)	
		Just right	39 (78)		38 (76)	
		Too few	0 (0)		9 (18)	
	**Agreed or strongly agreed with the statements, n (%)**					
		“The text messages helped me quit smoking”	47 (94)		40 (80)	
		“I learned a lot from using the text program”	48 (96)		36 (72)	
		“The text program gave me confidence to quit”	43 (86)		40 (80)	
		“The text messages motivated me to quit smoking”	47 (94)		41 (82)	
		“Using the text program helped with cravings and triggers”	41 (92)		35 (70)	
		“Using the text program motivated me to try to quit again if I quit and then started to smoke again”	45 (90)		37 (74)	
		“I trusted the information in the messages”	49 (98)		—	
		“The text messages gave me ideas about how to refuse cigarettes offered by others”	41 (92)		—	

^a^The program refers to the SMS cessation program for the intervention arm and weekly text assessment for the control arm.

^b^Not applicable.

^c^Among participants who had ever texted keywords to trigger on-demand messages (n=19).

^d^Among participants who had ever responded to text assessment (n=36).

### Acceptability

All but one participant in the intervention arm reported being satisfied or very satisfied with the program overall and none reported being very unsatisfied (Table 2). In contrast, none of the participants in the control arm reported being very satisfied and 18% (9/50) reported being very unsatisfied. The majority of participants in both arms perceived the number of messages as just right, however 18% (9/50) of participants in the control arm responded that there were too few compared with none in the intervention arm. Although for both arms there was a high level of agreement that the program increased confidence, was helpful, and increased motivation, participants in the intervention arm consistently expressed higher levels of acceptability across these measures than those in the control arm.

### Smoking Abstinence, Quit Attempts, and Reduction in CPDs

The biochemically verified abstinence was higher in the intervention arm than the control arm (6 week: 20% [10/50] vs 2% [1/50]; *P*=.01; 12 week: 12% [6/50] vs 6% [3/50]; *P*=.49), although the difference was not significant at 12 weeks (Table 3). The proportion of participants who reported quit attempts increased from 6 weeks to 12 weeks in both arms, but we observed no difference between the two arms. Similarly, the reduction in CPD increased over time but there was no difference by arm.

**Table 3:** Abstinence outcomes at 6-week and 12-week follow-up.

Measure		Intervention (n=50)	Control arm (n=50)	*P* value
**6-week follow-up**				
	Biochemically verified abstinence, n (%)	10 (20)	1 (2)	.01
	Quit attempt, n (%)	21 (68)	28 (68)	>.99
	Reduction in CPD^a^ as compared to baseline^b^, mean (SD)	9.3 (7.8)	6.8 (5.8)	.13
**12-week follow-up**				
	Biochemically verified abstinence, n (%)	6 (12)	3 (6)	.49
	Quit attempt, n (%)	27 (79)	32 (87)	.53
	Reduction in CPD as compared to baseline^b^, mean (SD)	11.1 (8.3)	9.9 (7.3)	.52

^a^CPD: cigarette consumption per day.

^b^Among participants who reported not quit yet.

### Qualitative Findings

The qualitative data supported and expanded on the survey findings for the intervention arm. The main themes that emerged included the overall value of the SMS program, message preferences, perceptions about specific features (eg, timing, bidirectional messaging), and recommendations for enhancing the program. Almost all of the participants liked the SMS program and described it as helpful, primarily because it offered encouragement and enhanced motivation and served as a reminder to stay on track.

The messages motivated me and reminded me not to smoke.

#15, 39 years, male

I felt like they [text messages] made me determined to quit.

#16, 43 years, female

Messages that included craving management strategies and addressed the harmful effects of smoking were described as particularly useful. A participant noted that he “learned many things…about the ways to overcome cravings” [#1, 54 years, male]. Another explained that messages about the negative consequences of tobacco were “like a warning, helping us understand the danger of smoking and benefits of quitting. So we became conscious and then decided to quit” [#8, 50 years, male].

Reactions to messages that suggested strategies for refusing cigarettes in social situations were mostly positive. One participant noted that those messages “provided the most simple and effective way to refuse invitations to smoke” [#10, 50 years, male]. However, a few participants suggested rewording these in ways that were more consistent with how they communicate with friends and family.

Many of the participants preferred a more tailored approach in terms of message timing.

You should send text messages to the relevant time frame of each individual.

#6, 34 years, male

One participant wanted the messages to be sent when he had the cravings.

You could send the messages at those time.

#6, 34 years, male

Participants did have the option to proactively text the keyword TIPS to generate messages during those difficult times, but few routinely used the option.

Participants suggested several additional modifications to enhance the program. Additional content changes including adding more text messages about the health consequences of smoking.

I want to receive more text messages on the risks of smoking so my determination in quitting may be stronger.

#8, 50 years, male

Participants expressed a strong interest in a more interactive approach.

Sometimes I wanted to interact with the person who sent the messages, but I could not do that. The interaction was limited to the responses of yes or no.

#6, 34 years, male

Similarly, another suggested allowing users to “ask [text] my own questions” (#4, 51 *years*, male) rather than using the keyword. A few participants suggested adding telephone interactions with a counselor. Last, the majority of smokers suggested extending the intervention duration.

I want to receive text messages for a longer time.

#6, 34 years, male

[I]t may be more effective if the duration is longer.

#11, 33 years, male

## Discussion

### Principal Findings

We found support for the feasibility and acceptability of a culturally and linguistically adapted SMS program developed for tobacco users in Vietnam. Smokers overwhelmingly agreed that the messages were helpful, motivated them to quit, and that they would continue to use the program if it was available. A majority of those in the intervention arm also reported that they usually or always read the messages. However, few participants took advantage of the interactive feature that offered them the opportunity to elicit additional support by texting the keyword. Qualitative data suggested that Vietnamese smokers preferred to receive tips as part of the main program and to interact on an as needed basis in a way that would allow them to ask questions and receive tailored responses.

Engagement, defined as the mean number of days that participants used the program, was relatively high compared to previous studies. In the intervention arm, 60% remained in the program for at least 5 weeks. This is in contrast to studies in high income countries that have reported challenges retaining SMS participants [22]. This may be related to the novel nature of this type of intervention in Vietnam. Additional modifications to the program design, as suggested by the participants, may further increase engagement.

The need for significant changes in content and tone of the original message libraries demonstrated the importance of local adaptation in LMICs. As an example, the original message libraries included very few messages about the dangers of tobacco use and instead focused on the benefits of quitting. In contrast, at each stage of development, smokers expressed a preference for more messages that used negative framing of health risks (eg, “If you continue to smoke, your risk of dying from cancer is 25% higher than nonsmokers”). A review of studies that analyzed the impact of emphasizing benefits (gain framed) versus costs (loss framed) found a small advantage of gain versus loss framed messages, but findings were mixed, and these data are based on studies in high income countries [31]. In the process of adapting programs to LMIC contexts, there is an opportunity to continue to explore how messages can be more effectively designed to motivate long-term abstinence. Whether gain or loss framed, an emphasis on health risks seems important in LMIC contexts.

The control arm’s high level of engagement and satisfaction with the program that only included weekly text assessments was an unexpected finding. This may reflect the lack of prior experiences with any smoking cessation services among Vietnamese smokers. Vietnam has not widely disseminated WHO guidelines for integrating routine tobacco use screening and brief advice into the primary care health system [6], and although there is a Quitline, smokers were largely unaware of this resource. Hence, receiving even a weekly text question about their smoking status may have generated the perception that they were receiving tobacco cessation support. Despite their overall satisfaction, compared to the intervention arm, the control arm was less likely to achieve biochemically verified abstinence at 6 weeks. This provides some support for the SMS program’s specific content.

These results should be interpreted as preliminary given the small sample size of this pilot study, as well as the relative brevity of our program as compared to some of the existing SMS cessation interventions [19,20,32,33]. Participants were interested in engaging for longer durations, which could increase abstinence rates over time. However, the results are consistent with one of the few studies conducted in an LMIC. For example, in China, Liao et al [34] conducted a 12-week SMS cessation intervention and reported higher biochemically verified abstinence in intervention groups (high frequency message group: 6.5%; low-frequency message group: 6.0%) than the control group (1.9%). A recent review of mHealth cessation interventions in LMICs concluded that more rigorous studies, with longer follow up and biochemical verification, are needed to further study efficacy in LMIC [13].

Finally, the review by Krishnan et al [13] also suggested the need to compare different characteristics of mHealth cessation interventions. For example, participants requested more message tailoring. Tailoring messages to readiness may increase program effectiveness but findings from studies using this approach are not definitive [15,35]. A few studies outside of LMIC settings have also tested and reported promising findings for strategies that combine SMS programs with interpersonal supports such as peer mentoring from former smokers [16], individual counseling led by professional [35], and counselors’ responses to user composed questions [32]. Adding interpersonal supports may improve user experience and engagement [16,32,35,36] and was requested by our participants. However, the costs of integrating this format into automated SMS programs may reduce the feasibility of scaling programs nationally [37]. New approaches such as the use of automated chatbots may address one of the recommendations from participants to create opportunities for more human interaction with marginal costs. Chatbots offer a conversation interface that can both answer questions posed by the user in a natural language and ask them questions creating a virtual coach experience [38].

### Limitations

Our study has limitations. First, participants were recruited from two urban wards in one city. Thus, the findings may not be generalizable to all smokers in Vietnam. In addition, we excluded waterpipe-only smokers. Additional research is needed to explore the value of tailoring mHealth programs to specific types of tobacco users. Second, we did not conduct qualitative interviews with the control arm, which could have provided additional insights into their experience and reasons for the high levels of engagement and acceptability of weekly text assessment. Third, while the study sample was small, a strength of the study was the high retention rates, with all participants completing both follow-up assessments. Last, our study sample included only 2 women, which is consistent with the low smoking rate among Vietnamese women (1.1%) as smoking is less acceptable among women [39,40]. This may limit our ability to generalize to this population of smokers.

### Conclusion

Despite these limitations, this study contributes to the small body of literature on mobile phone smoking cessation treatment carried out in LMICs. The data supported the feasibility and acceptability of a culturally adapted SMS cessation treatment program and demonstrated short-term efficacy in promoting abstinence among Vietnamese smokers. While ongoing and future research continues to grow the evidence for effective mHealth cessation programs in a given context, LMICs are beginning to adopt and scale these programs [41]. Therefore, it is equally important to support the design and integration of low-cost monitoring and evaluation systems to guide program modifications that respond to user feedback and sustain and enhance program impact [42].

## Appendix

Multimedia Appendix 1 CONSORT-eHEALTH checklist (V 1.6.1).

## Abbreviations

CPD: cigarette consumption per day
LMIC: low- and middle-income country
mHealth: mobile health
NCI: National Cancer Institute
RCT: randomized controlled trial
SMS: short message service
WHO: World Health Organization
